# Evaluation of *in vitro* and *in vivo* Glycemic Index of common staples made from varieties of White Yam (*Dioscorea rotundata*)

**DOI:** 10.3389/fnut.2022.983212

**Published:** 2022-09-09

**Authors:** Toluwalope Emmanuel Eyinla, Rasaki Ajani Sanusi, Busie Maziya-Dixon

**Affiliations:** ^1^Food and Nutrition Sciences Laboratory, International Institute of Tropical Agriculture, Ibadan, Nigeria; ^2^Department of Human Nutrition and Dietetics, College of Medicine, University of Ibadan, Ibadan, Nigeria

**Keywords:** yam, glycemic carbohydrates, starch, diabetes, starch digestibility

## Abstract

Consumption of high Glycemic Index (GI) foods is a risk factor for increasing prevalence of diabetes mellitus (DM). The extent of variation in starch digestibility and GI of Yam varieties and products is not yet fully understood. This study was therefore designed to evaluate *in vitro* and *in vivo* Glycemic Index properties of commonly consumed products prepared from varieties of White Yam. Four products (boiled, fried, pounded yam, and *Amala*) were prepared from 5 common varieties of Yam and evaluated for Digestible Starch (DS) and Resistant Starch (RS). Based on results, two products-pounded yam and *Amala*-were processed from three of the most popular varieties. Analysis of Rapidly Digestible Starch (RDS), Slowly Digestible Starch (SDS), and estimated *in vitro* GI (eGI) were then carried out in this stage. Glycemic Index (GI) of these products consumed by apparently healthy young adults were also determined. Variety *Amula* had highest DS in *Amala* (19.1/100 g) and pounded Yam (20.4/100 g) while variety *Alumaco* had highest RS in all the products (2.9–3.3/100 g). When compared with RS in its raw tuber, RS of *Alumaco* generally increased after processing. Variety *Alumaco* had lowest RDS in Amala (0.6/100 g) and pounded yam (0.3/100 g) while eGI was lowest in *Alumaco* made into *Amala* (53) and pounded yam (48). Assessment of GI resulted in high GI for all products across each variety. Irrespective of variety, processing Yam into *Amala* released RDS fraction faster when compared with pounded yam. Generally, even though the products are considered as having high GI, *Amala* raised eGI and GI faster than Pounded yam. Variety-*Alumaco* particularly showed favorable properties applicable to dietary management of diabetes. Exploring more processing methods and genetic diversity is recommended.

## Introduction

Global rates of prevalences of non-communicable diseases such as diabetes mellitus have been reported to be on the increase even in African countries where problems of undernutrition still persist (1, 2). One of the major risk factors related to this high prevalence is the consumption of high carbohydrate content foods especially when it forms the bulk of dietary source of energy (3). Among diabetic patients including individuals applying body weight control diet plans, there is usually a careful selection of carbohydrate rich foods. A long-standing *in vivo* metric for measuring and managing blood glucose control in diabetics and healthy individuals is the Glycemic Index (GI) which is derived by comparing blood glucose response caused by a food/meal against a similar estimation of a reference food item, which could be glucose or white bread (4, 5).

In recent times, several studies have shown a significant association between the time taken for starch to digest and the glucose released using various *in-vitro* methods that simulate the popular *in vivo* GI methodology (3, 6). This rests on the discoveries that Carbohydrates which were historically classified as simple and complex fractions can now be categorized based on their digestibility and physiological impact as food passes through the gastrointestinal tract. Accordingly, mono- and disaccharides and rapidly or slowly absorbed starches are now categorized to be glycemic carbohydrates (7). Thus, measurement of physiological effects by categorizing foods based on their glycemic effects are now providing useful guidelines for managing morbidity and preventing mortality in diabetic individuals (6).

The rate of starch digestibility still depends largely on the agronomic origin of the food which influences starch structure, starch gelatinization after processing quantity, type of resistant starch and dietary fiber (8, 9). Yam-a major food security crop for Sub-Saharan Africa-is the second most produced root and tuber crop after cassava (10, 11). Yam (*Dioscorea* spp.) has a large biological diversity with more than 600 species worldwide but only six are widely cultivated in Sub-Saharan Africa as follows: *D. alata, D. bulbifera, D. dumetorum, D. esculenta, D. cayenensis*, and *D. rotundata* (11). Of them all, the most popularly cultivated of all is White Yam (*D. rotundata*) of which about 73 million tons were produced in 2020 with about 70% coming from Nigeria (10).

In general, existing literature, points to a variation in digestibility of starch and GI due to processing method, structure, or origin of plant material. The GI of foods (and in some cases their starch digestibility) has been established as a tool for managing abnormal blood glucose levels, but the existence of biodiversity in commonly consumed starchy staples with the purpose of influencing differing post-prandial blood glucose levels is not yet fully explored. While published studies have established the possible intra-varietal differences in GI and starch digestibility (using *in vitro* methods) of some common products of Cassava (12), Sweet Potato (13), Yellow yam (14, 15), there is still little information on possible variation among common products of different varieties of White Yam commonly cultivated in Nigeria. This study therefore sought to evaluate *in vitro* and *in vivo* Glycemic Index properties of commonly consumed products prepared from some varieties of Yam.

## Materials and methods

### Study design

The study combined laboratory experiments and a human feeding experiment carried out in three stages. The first stage involved food compositional analysis of products of selected yam varieties. In stage two, digestible and resistant starch fractions were evaluated. In the final stage, three varieties (and two products) were analyzed for rapidly and slowly digestible starch fractions, estimated glycemic index (eGI), and glycemic index (GI).

### Source of materials

A literature and database search of yam varieties cultivated in Nigeria was carried out which was shortlisted to 5 most popular varieties in consultation with the Yam Breeding Unit of the International Institute of Tropical Agriculture (IITA) Ibadan, Nigeria (16). The varieties (*Meccakusa*, TDr02665, *Alumaco, Oju Iyawo, Amula*) were grown under rain-fed conditions with no fertilizer or herbicides added.

### Preparation of products

#### Preparation of boiled yam

The peeled and washed samples were reduced into small sizes of about 12 cm diameter and boiled in water at 100°C for 20–30 min (17).

#### Preparation of pounded yam

Washed yam slices were boiled in distilled water and pounded with a mortar and pestle until a constant dough was obtained (17).

#### Preparation of fried yam

Yam tubers were peeled and sliced. Washed yam slices were deep fried in vegetable oil at constant heat of 180°C for 5 min. Small quantity of water was added at intervals so the sample could uniformly cook and also to reduce the evaporation of the frying oil (17).

#### Preparation of amala

Tubers were peeled and washed with distilled water but not sliced further before being parboiled at 60°C for 12 min, and soaked in water for 18 h. The slices were then dried in the sun for 3–4 days. After which it was ground to flour (*elubo*), and cooked into a thick dough (17).

### Stage 1: Food compositional analysis

Moisture, protein, fat, dietary fiber and ash content were determined using the methods described previously (12, 18). Carbohydrate was determined by difference. The moisture content was determined through drying for 16 h at a constant temperature 103°C (18). The protein content was determined based on method described in literature (19) which involved spectrophotometric determination after the food matrix is subjected to digestion using concentrated acid solution. Fat content was determined using an Automated Soxhlet extractor (FOSS Soxtec^TM^ 8000) which used hexane as the extracting solvent. Dietary fiber was determined gravimetrically after subtraction of digested fat and protein content as described in (12, 16, 20). Ash content was measured after the food sample was placed in pre-heated muffle furnace for 6 h at 550°C (18).

### Stage 2: Measurement of digestible and resistant starch

The evaluation of digestible and resistant starch fractions in raw tuber and products was done using methods described previously in literature (12, 20) which involved the initial process of hydrolysis and solubilization of starch in the yam product before the quantifications of the different fractions of starch. Hydrolysis was achieved by mixing an enzymatic solution with sample and incubating (with continuous shaking) for 16 h. Hydrolysis was stopped with addition of ethanol and the mixture was centrifuged. The supernatant was then separated from the mixture and evaluated for digestible (solubilized) starch while the remainder pellets were used to calculate non-solubilized (Resistant) starch. Mathematical calculations were done using the Megazyme Mega-Calc^TM^ which was made available as a spreadsheet application via www.megazyme.com.

### Stage 3: Measurement *in vitro* starch digestibility, eGI, and GI

#### Quantification of slowly digestible starch, rapidly digestible starch

The method and principles earlier reported (12, 16) were applied in this study. First a preparation of enzyme solution of Pancreatic α-amylase (0.45 g) and amyloglucosidase (AMG) was done. Secondly, the sample and a buffer solution of sodium acetate (pH 5.2) were incubated in a shaking water bath (37°C, 120 rpm). At the designated time intervals, aliquots were taken from the incubating mixture for glucose determination using a Glucose reagent kit (supplied by Megazyme, Ireland, UK). The rate of starch digestion was expressed as the percentage of total starch hydrolyzed at different times (0, 10, 20, 30, 60, 90, 120, and 180 min). The total starch hydrolysis (%) which was a fraction of glucose (converted to starch) released per time was calculated and classified as follows:

1. Rapidly digestible starch (RDS, digested within 20 min).
2. Slowly digestible starch (SDS, digested between 20 and 120 min).

#### Calculation of estimated glycemic index (*in vitro* method)

The method described (21) was applied to extrapolate the first order kinetic equation: C = C (1 - e-kt), where C represented the percentage of starch hydrolyzed at time t (min), C is the equilibrium percentage of starch hydrolyzed after 180 min, and k is the kinetic constant. The parameters, C and k, were estimated for each product based on the data obtained from the *in vitro* starch digestion. The hydrolysis index (HI) was then derived by dividing the Area Under Curve (AUC) of each starch hydrolysis by the AUC of the reference food (white bread). The HI represented the rate of starch digestion and the eGI indicated the digestibility of the starch in relation to the digestibility of starch in the reference material. The pGI was then calculated using the equation presented and applied by previous studies (16, 21); eGI = 39.71 + 0.54HI.

#### Determination of GI (*in vivo* method)

The methods described in literature (5) were applied in determining GI of the food products. Apparently healthy participants (*n* = 33) were recruited and screened before being admitted to the procedure. The portion used for testing was 50 g of available carbohydrate both of the reference food item (glucose) and the food products. The experimental portions served were deduced from the values of digestible carbohydrates. Glycemic responses of each participant were obtained from capillary blood and used in calculating the incremental area under the glucose response curve (IAUC) after which GI was obtained by calculating the percentage area under the curve of the reference material (glucose) in comparison with the test food. The GI categorization of foods was based on low (<55), medium (56–69), high (>70).

### Data analysis and ethical considerations

Data obtained from the study was entered into spreadsheets before descriptive and inferential statistical procedures were applied to present the study's results with *p*-value set at α_0.05_. Mean, standard deviation, coefficient of variation and analysis of variance tests (one-way and two-way repeated measures) were applied on the study's results. Duncan's test was applied to separate mean of values. For all inferential statistics, significance was set at *p* < 0.05. Ethical guidelines including informed consent before blood collection were followed as prescribed by the University College Hospital/University of Ibadan ethical review committee where the study's ethical clearance was registered with approval number UI/EC/17/0407 (approved 28/11/2017).

## Results and discussion

### Food composition analysis

The nutrient composition of Boiled, fried, pounded yam and *Amala* are presented in Table 1. In boiled yam, the mean moisture content was 70.25 ± 3.58/100 g, while the total dietary fiber ranged from 2.34 to 5.21/100 g in varieties *Meccakusa*, and *Amula*, respectively. The coefficient of variation was highest in the ash contents of the products at 69.43% and lowest in moisture contents (5.09%). The average moisture content of pounded yam was 72.15/100 g while the carbohydrate content ranged from 19.41/100 g in *Oju-iyawo* to 24.12/100 g in TDr/02665. The total dietary fiber ranged from 2.53/100 g in *Meccakusa* to 4.46 g in *Alumaco* and the coefficient of variation was lowest in moisture content (2.62%). The mean moisture content of fried yam was 41.89 ± 7.32/100 g and the coefficient of variation was highest in the ash content of the products at 63.72%. The lowest ash content was found in *Amula* which had 0.88/100 g while TDr/02665 had 4.84/100 g which was the highest of all. *Amala* from selected Yam varieties had the highest mean moisture content of 77.12 ± 2.02/100 g when compared with other products. The carbohydrate contents ranged from 14.77 g in *Oju-iyawo* to 21.58/100 g in TDr/02665 while the total dietary fiber was from 2.69/100 g in *Alumaco* to 4.05/100 g in *Amula*.

**Table 1:** Nutrient composition of products (per 100 g) from varieties of Yam.

		**Moisture**	**Protein**	**Fat**	**Carbohydrate**	**Dietary fiber**	**Ash**
Boiled Yam	*Meccakusa*	73.40^bc^	1.44^bc^	0.35^ab^	19.07^a^	2.34^a^	3.40^c^
	TDr/02665	70.58^bc^	1.09^ab^	0.30^ab^	22.74^a^	2.40^a^	2.89^bc^
	*Alumaco*	67.04^ab^	1.52^bc^	0.84^c^	25.26^ab^	4.12^cd^	1.21^ab^
	*Oju Iyawo*	73.62^bc^	1.75^c^	0.68^bc^	19.98^a^	3.15^abcd^	0.81^a^
	*Amula*	73.06^bc^	0.58^a^	0.20^a^	21.82^a^	3.83^bcd^	0.52^a^
	Mean	70.25	1.26	0.43	23.23	3.26	1.56
	SD	3.58	0.32	0.24	4.12	0.63	1.09
	^**^CV	5.09	25.06	55.65	17.72	19.34	69.43
Pounded Yam	*Meccakusa*	71.97^b^	0.86^abc^	0.13^a^	23.74^bc^	2.53^ab^	0.77^abc^
	TDr/02665	70.95^ab^	0.98^abc^	0.21^abc^	24.12^bc^	2.59^ab^	1.14^c^
	*Alumaco*	70.98^ab^	1.03^abcd^	0.28^abcd^	22.16^ab^	4.46^d^	1.08^bc^
	*Oju Iyawo*	76.06^c^	0.97^abc^	0.21^abc^	19.41^a^	2.69^ab^	0.67^a^
	*Amula*	68.29^a^	1.55^d^	0.42^d^	25.34^c^	3.65^bcd^	0.75^ab^
	Mean	72.15	1.08	0.26	22.64	3.13	0.73
	SD	1.89	0.28	0.09	1.52	0.66	0.21
	^**^CV	2.62	25.59	36.01	6.71	21.12	29.02
Fried Yam	*Meccakusa*	38.26^ab^	1.58^ab^	3.98^d^	50.22^bc^	2.02^ab^	3.95^bc^
	TDr/02665	54.50^d^	1.12^a^	3.62^cd^	33.40^a^	2.51^abc^	4.84^c^
	*Alumaco*	45.34^bcd^	1.92^abc^	3.32^abcd^	44.29^abc^	3.50^bc^	1.63^ab^
	*Oju Iyawo*	37.24^ab^	1.99^abc^	2.69^ab^	54.26^c^	2.16^ab^	1.67^ab^
	*Amula*	43.77^bcd^	1.40^a^	2.52^a^	48.29^bc^	3.14^bc^	0.88^a^
	Mean	41.89	1.90	3.19	48.33	2.55	2.13
	SD	7.32	0.59	0.43	7.66	0.80	1.36
	^**^CV	17.48	30.87	13.34	15.85	31.31	63.72
*Amala*	*Meccakusa*	75.47^a^	1.04^b^	0.29^abc^	18.57^bc^	3.82^c^	0.80^c^
	TDr/02665	75.12^a^	0.38^a^	0.19^ab^	21.58^c^	2.10^ab^	0.63^abc^
	*Alumaco*	77.04^ab^	0.77^ab^	0.21^ab^	18.92^bc^	2.69^bc^	0.37^a^
	*Oju Iyawo*	80.30^bc^	1.04^b^	0.63^bcd^	14.77^ab^	2.79^bc^	0.47^ab^
	*Amula*	80.97^c^	0.29^a^	0.08^a^	14.19^a^	4.05^c^	0.42^a^
	Mean	77.12	0.74	0.35	18.48	2.78	0.52
	SD	2.02	0.27	0.24	2.35	0.80	0.14
	^**^CV	2.62	35.80	68.61	12.74	28.85	26.54

** Percentage Coefficient of variation.

Superscripts represent significance and separation of mean for nutrients across columns.

Values are mean of duplicates.

The statistical measures of coefficient of variation and means separation as applied in this study establishes that there are differences in the selected varieties. This was similarly found by Kouassi and et al. (15) who evaluated different varieties of yellow yam (*Dioscorea cayenensis*). Worthy of note in line with the study's objective to probe carbohydrate properties is the differences found with dietary fiber composition which is scarce. There is sufficient evidence that dietary fiber has potentials to provide short and long-term functionality to the gastrointestinal tract (22). Even though the variations in the total dietary fiber among varieties were not substantial, this study found an effect of processing on this nutrient. However, the impact on dietary intake and nutrient adequacy may be marginal especially when compared with values obtainable from local and regional food databases (23, 24). Albeit, this study still successfully highlights the different variations in composition that could be exploited for breeding purposes or food science applications.

### Digestible and resistant starch

**Figure 1:**
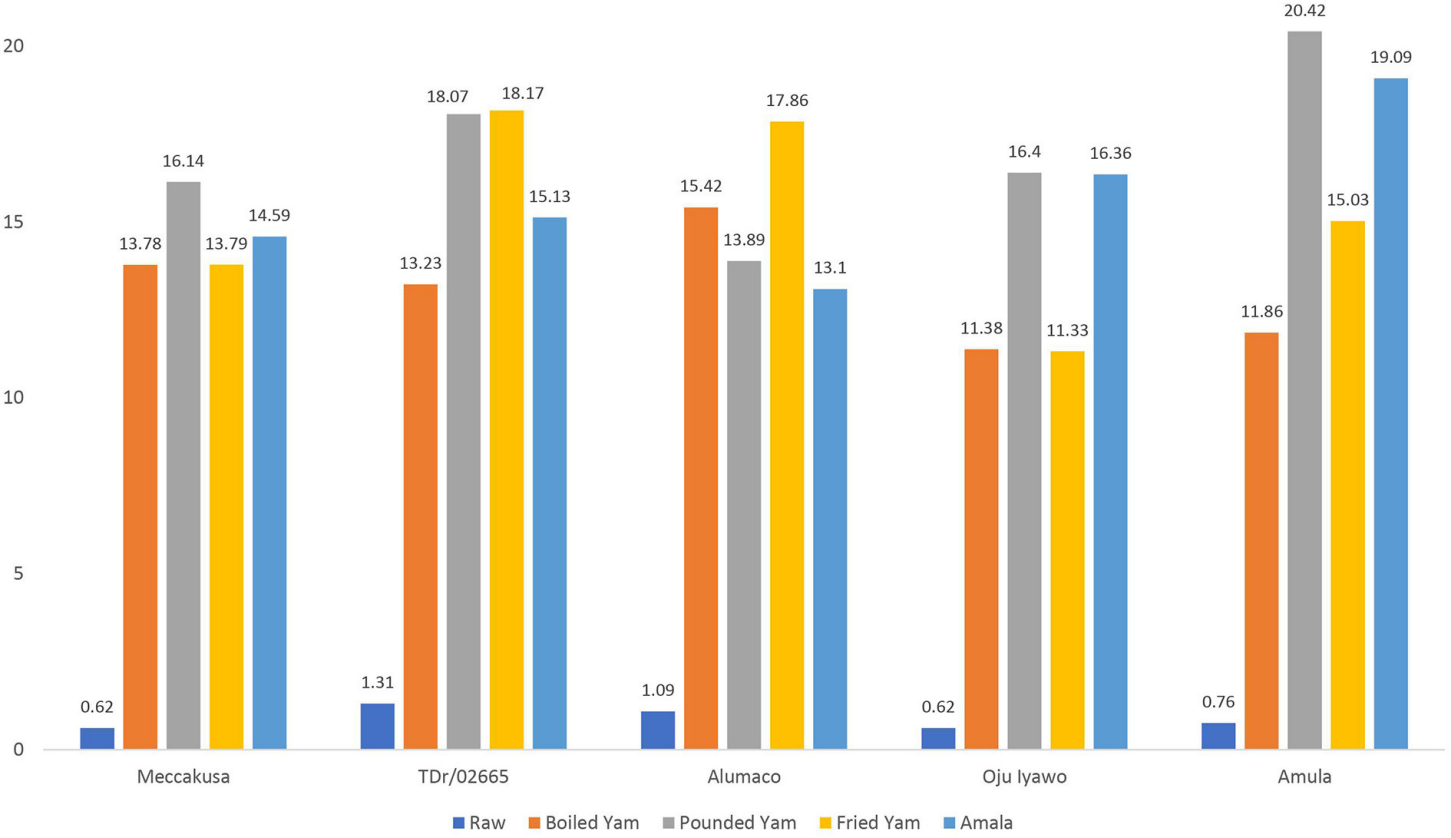
Digestible Starch of raw tuber and products from five selected yam varieties.

**Figure 2:**
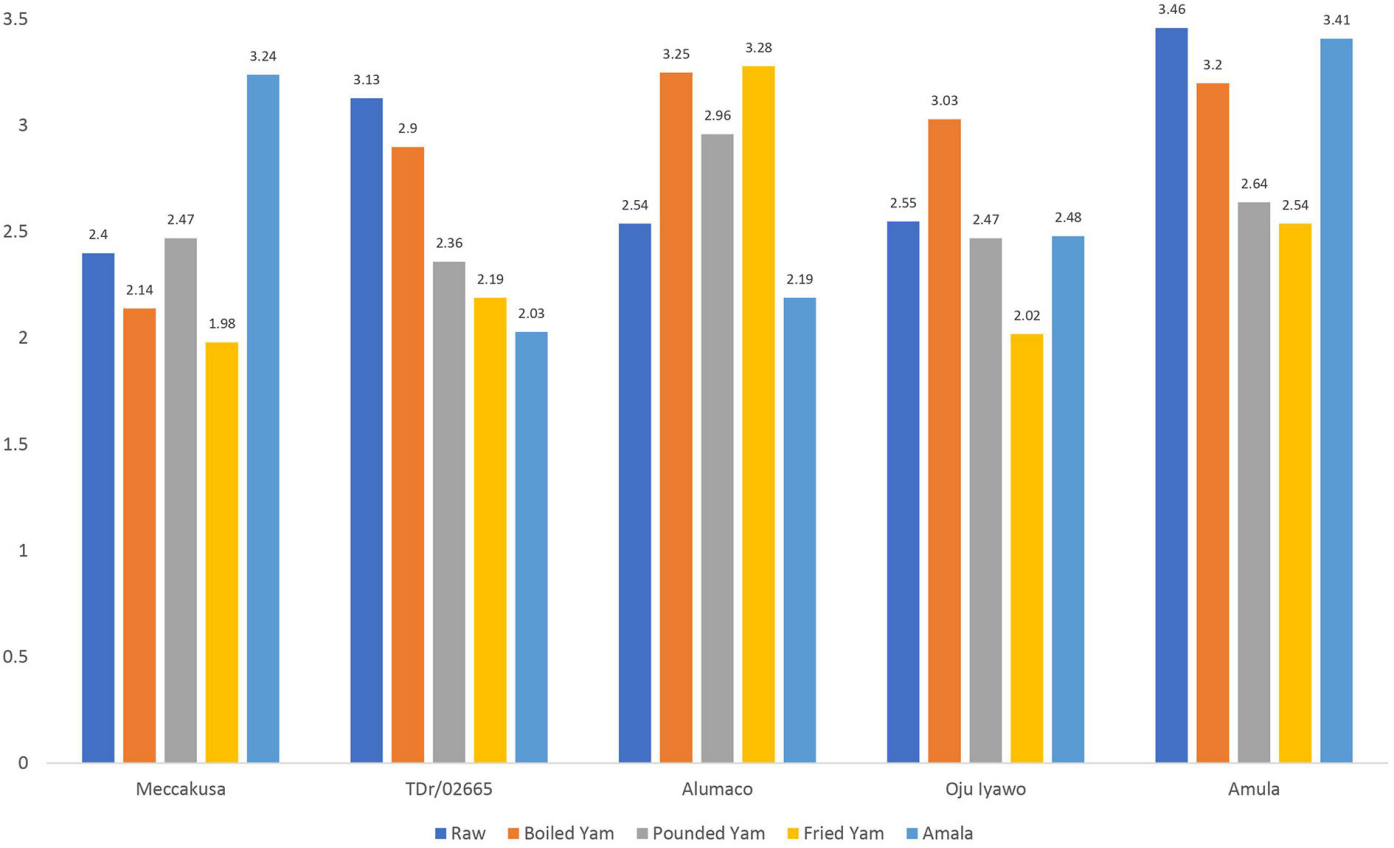
Resistant Starch of raw tuber and products from five selected yam varieties.

Figures 1, 2 depicts the digestible and resistant starch fractions in the raw tuber and four products processed from five varieties of yam as considered in this study. The variety with the highest digestible starch when processed into boiled yam was *Alumaco* (15.42/100 g) while the lowest was found in *Oju-iyawo* (11.38/100 g). A comparison between digestible starch in raw tubers and processed boiled yam shows that the highest percentage increase in digestibility is shown in *Meccakusa* (95.50%) while the lowest was in TDr 02665 which showed a 90.10% increase compared with the raw tubers. The variety with the highest content of digestible starch and digestibility when processed into pounded yam was *Amula* (20.42/100 g) with digestibility of 96.28% while the lowest was found in *Alumaco* (13.89/100 g) at 92.15%. When the raw tubers were processed into fried yam, digestible starch was highest in TDr 02665 (18.17 g) and was lowest in *Oju-iyawo* (11.33/100 g). The highest percentage increase in digestibility results for *Amala* was shown in *Amula* (96.02%) and *Oju-iyawo* (96.02%) while the lowest was in TDr 02665 which showed a 91.34% increase compared with raw tubers. In boiled yam, the variety with the highest content of resistant starch per 100 g after processing was *Meccakusa* (3.24/100 g) while the lowest was 2.19/100 g in *Alumaco* and TDr 02665 which contained 2.03/100 g.

The highest depletion in resistant starch in pounded yam occurred in TDr 02665 (24.60%) while the highest increase was in *Alumaco* which showed a 25.46% increase compared to the resistant starch in their respective raw tubers. The variety with the highest content of resistant starch per 100 g after processing into pounded yam was *Alumaco* (2.96 g) while the lowest was 2.36/100 g in TDr 02665. After processing into fried yam, the highest depletion in resistant starch was in TDr 02665 (30.03%) while the highest increase was in *Alumaco* which showed a 29.13% increase compared to the resistant starch content in its raw tubers. As for depletions or increment in resistant starch content after processing into *Amala, Meccakusa* (10.83%) was depleted the most while the highest increase was in *Alumaco* which showed a 27.95% increase compared to the resistant starch in raw tubers.

The similar proportion of increased digestible starch across the four products considered in this study confirms the known fact that disrupting the food matrix from its raw state into consumable forms will increase digestibility. Ahmed and Urooj (25) presented results supporting this fact in a trial of cooking *Dioscorea alata* tubers using different methods. A test of between-subjects effect of DS as influenced by yam variety and product (**Table 3**) also revealed no statistical difference while there was an observed significance for RS. The importance of the resulting fractions (digestible and resistant) to the rate of carbohydrate digestion has been reviewed (7, 26) but more emphasis has been placed on RS fractions due to their physiological and nutritional relevance during carbohydrate metabolism (20, 22). In general, foods with significant amount of resistant starch have been proven to contribute not only to controlling the release of glucose post-prandially but also to colonic health since they constitute soluble dietary fiber that is beneficial in the large intestine. A distinct varietal attribute was observed in the resistance of starch in the yam products considered in this study. This distinction (asides from the general depletion of RS after processing) was an increase in resistant starch content of some of the products made from variety *Alumaco* which had an increase in RS contents with processing. This attribute places this variety in a class of its own against the expected depletion of resistant starch with processing and can be further explored for application in the control of blood glucose among diabetic individuals. The product that resulted in a substantial decrease in resistant starch after processing was fried yam which implies it will result in faster glucose release post-prandially. A similar impact of frying on food matrix was reported in a study that evaluated different Yam species and cooking methods whereby frying resulted in highest glycemic response (GI) compared to boiling or roasting (14). This attribute seems to be peculiar to fried products which undergo high heat preparation, since a similar observation in French fries (from potatoes) has been reported (13, 27).

### Rapidly digestible starch and slowly digestible starch, estimated glycemic index and glycemic indices

Starch digestibility results from *in vitro* analysis using the parameters- rapidly and slowly digestible starch and eGI are presented in Table 2 with *in vivo* GI results. Results for Pounded Yam showed that *Amula* had the highest SDS fraction (20.08/100 g). *Oju-iyawo* had a slightly higher RDS (0.36/100 g) in comparison with the other two varieties considered. The values of both RDS and SDS fractions were similarly lowest in *Alumaco* at 0.34/100 g and 13.55/100 g, respectively. In *Amala, Amula* had the highest slowly digestible starch fraction (17.84/100 g). *Oju-iyawo* had the highest rapidly digestible starch (1.38/100 g) in comparison with the other two varieties. Despite the similarities in rapid and slowly digestible starches of pounded yam and *Amala*, when the Yam was processed into *Amala*, it released RDS fraction faster when compared to pounded yam. This is explainable due to the impact of processing which is obvious by the steps required to obtain *Amala* in which boiling, drying, blending to flour and then cooking to dough will disrupt starch structure more when compared with preparing pounded yam. The method of processing pounded yam which involves a pounding effort that increases cohesiveness in the texture of the final product could also be responsible for its resistance to starch hydrolysis. In a study of Chinese yam (*Dioscorea opposita*), a similar inhibitory character was attributed to starch-protein interaction in the food matrix (28) while another study reported enzymatic inhibition (29).

**Table 2:** ^*^Rapidly Digestible Starch (RDS), Slowly Digestible Starch (SDS) and GI values in *Amala* and Pounded Yam made from Yam (g/100 g).

	**Variety**	**RDS**	**SDS**	**Experimental portion (g)**	**Mean GI**	**GI category**	**eGI**
*Amala*	*Oju Iyawo*	1.38^b^	14.98^b^	261.96	86^a^	High	59.39^a^
	*Amula*	1.25^ab^	17.84^c^	305.66	89^a^	High	57.78^a^
	*Alumaco*	0.61^a^	12.49^a^	381.67	83^a^	High	52.99^a^
Pounded Yam	*Oju Iyawo*	0.36^a^	16.04^ab^	304.84	92^a^	High	48^a^
	*Amula*	0.34^a^	20.08^c^	244.81	97^a^	High	49^a^
	*Alumaco*	0.34^a^	13.55^a^	359.85	104^a^	High	48.2^a^

GI, Glycemic Index; eGI, estimated Glycemic Index.

* Values represent mean of duplicates with exception of GI which represents mean of 11 participants.

Different superscripts represent significance and separation of mean for across columns for *Amala* and Pounded Yam, respectively.

**Figure 3:**
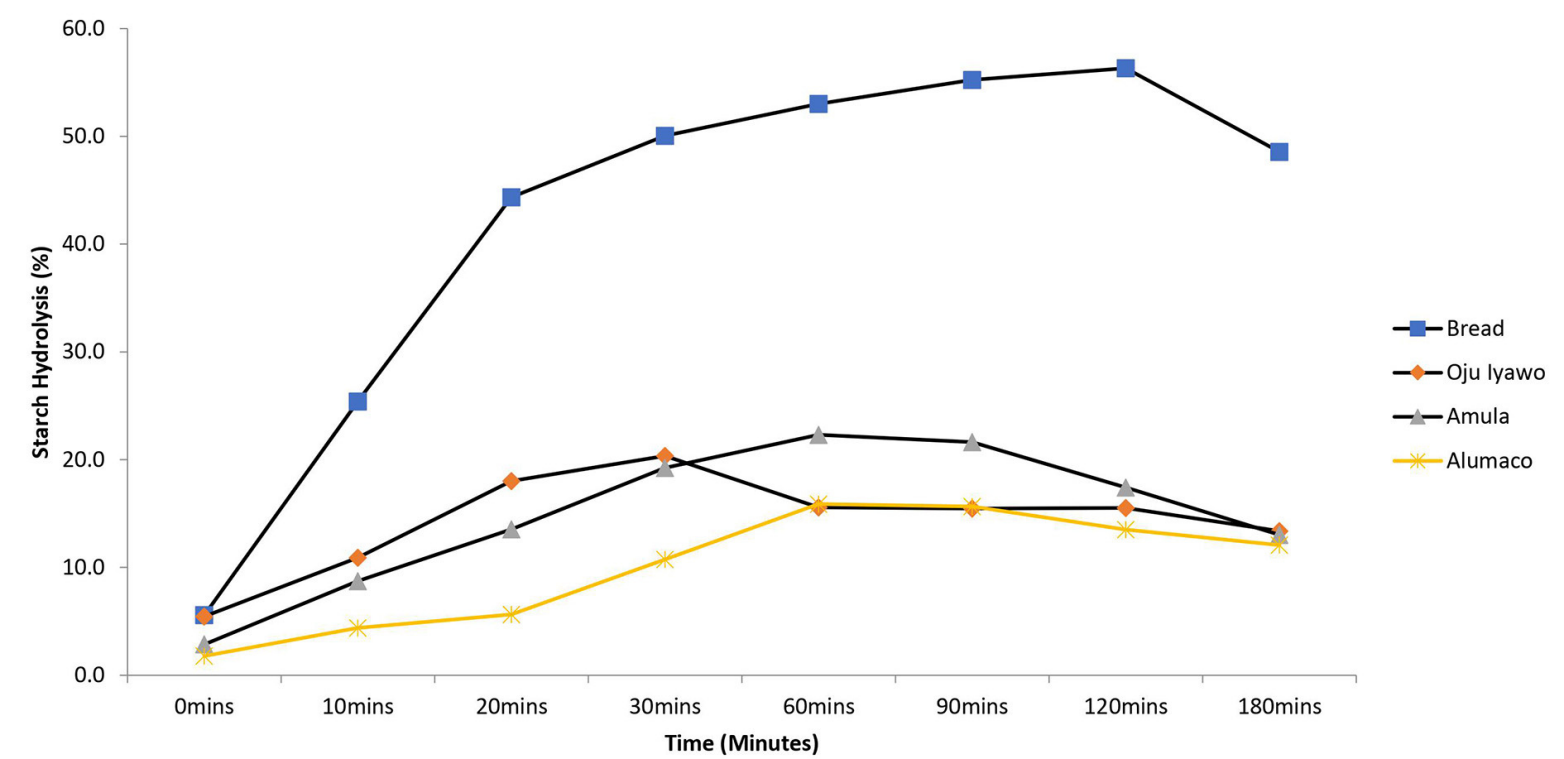
Rate of *in-vitro* Starch Hydrolysis in *Amala* made from three varieties of Yam.

**Figure 4:**
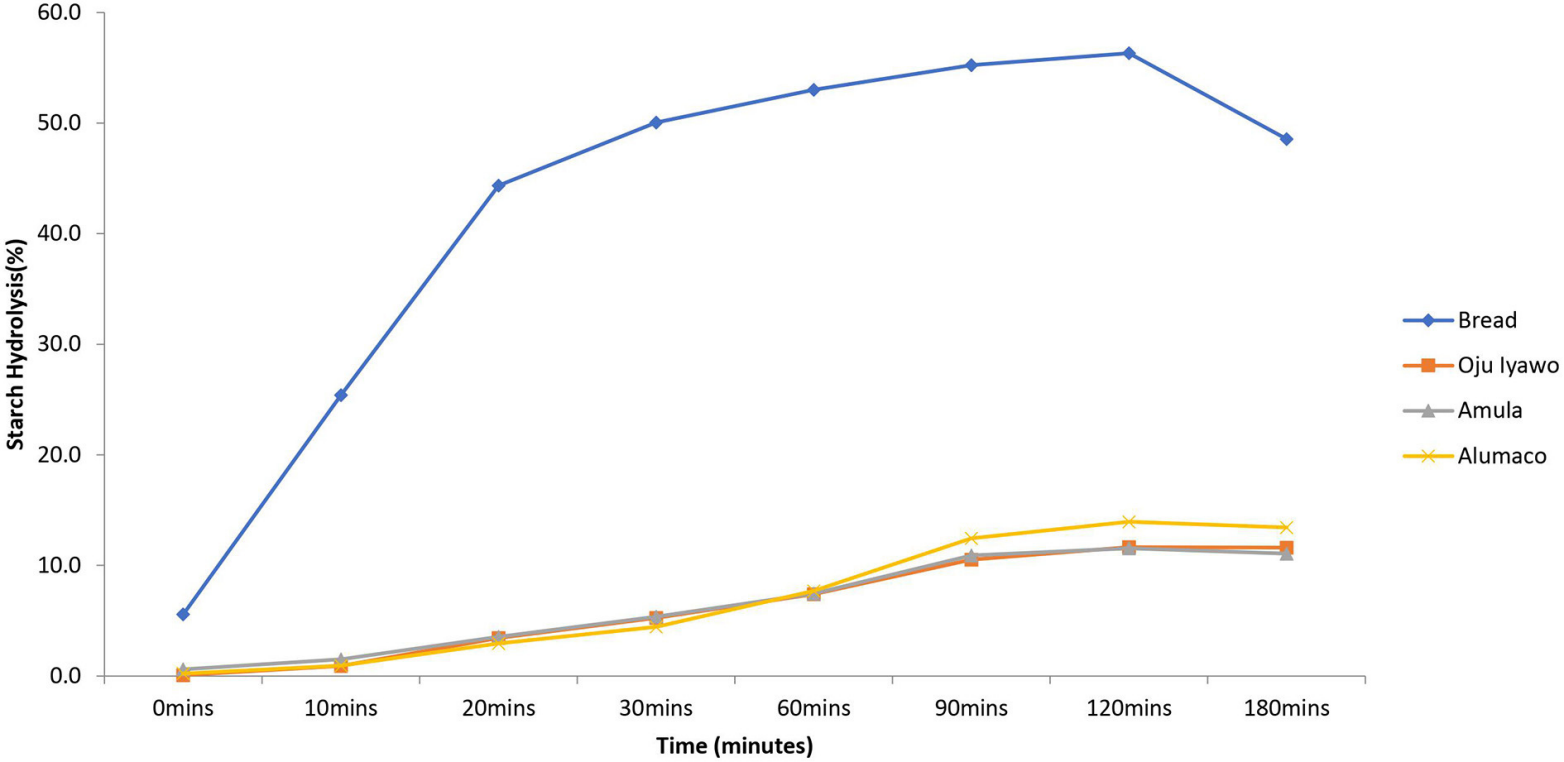
Rate of *in-vitro* Starch Hydrolysis in Pounded yam made from three varieties of Yam.

When starch hydrolysis was compared using the reference food material (white bread) as a basis, the *Amala* prepared from *Oju-iyawo* had the highest starch hydrolysis rate after 30 min compared to the other two varieties (Figure 3). Beyond 30 min *Amula* had a slightly higher rate of hydrolysis compared with the other varieties while *Alumaco* had the lowest rate of hydrolysis over the total time of hydrolysis. These hydrolysis rates resulted in eGI of 59, 57, and 53 for *Oju Iyawo, Amula*, and *Alumaco*, respectively. Compared to *Amala*, the rate of hydrolysis in pounded yam (Figure 4) was not pictorially distinct across each variety but was similar to that of *Amala* whereby variety *Amula* marginally had highest hydrolysis rates across 180 min while Alumaco had the lowest rate. These rates resulted in eGI of 48, 49, and 48 for *Oju Iyawo, Amula*, and *Alumaco*, respectively. The low gradient curves in comparison to white bread (more pronounced in pounded Yam) seems to be an attribute of yam species due to an increased degree of α-amylase and α-glucosidase inhibition action when its flours are processed into dough (29). All the products made from the yam varieties were in the high category of GI with *Amula* having highest GI of 89 in *Amala* and *Alumaco* (104) in pounded Yam. The results of the *in-vivo* assessments presented in this study were similar to a compilation of GI literature values which confirms Yam in most of its different food forms and varieties will result in high GI after consumption (14, 15, 30). Despite all products falling in the high GI category (>70), the numerical differences found with each product show that variety and cooking method can impact GI in Yam (14, 15). In comparison with literature values these differences may be attributed to variations in human responses and method or tools used to estimate the glycemic response (4).

Overall, despite the similarities in quantitative values, there was a statistical difference across the RDS, SDS, GI and eGI values across both varieties and products, respectively (Table 3). This distinction in each variety and product were more pronounced when the products were subjected to *in vitro* analysis than results gotten from the *in vivo* test. The main varietal distinction was observed in *Alumaco* which resulted in the lowest rapidly digestible starch across the two products. Real life applications of these findings will include dietary management of diabetic patients and also an advantage over the use of GI which can hardly be used to distinguish between varieties in this manner. Another observable trend in the results is that the higher/lower the content of rapidly digestible material in the food, the more glucose is released into the blood which translated into a higher/lower eGI. This suggests that there are stronger associations between the glycemic parameters (such as rapidly/slowly digestible starch, dietary fiber and resistant starch) with eGI and GI compared to proximate parameters such as fat, ash and protein. This assertion was also suggested by Arvidsson-Lenner et al. (22) and Afandi et al., (31) who reported that variation in digestibility properties is more reliant on resistant starch composition and the granular structure of the starch.

**Table 3:** Test of between-subjects effect of *in vitro* and *in vivo* parameters as influenced by yam variety and product.

	**RS**	**DS**	**RDS**	**SDS**	**eGI**	**GI**
Variety	0.010	0.053	0.000	0.000	0.039	0.014
Product	0.003	0.791	0.000	0.000	0.000	0.000
Variety × Product	0.005	0.995	0.000	0.000	0.044	0.001

## Conclusion

This study evaluated the differences in nutrient compositions, starch digestibility properties and GI of common staples made from Yam varieties across three stages of experiments. Overall, there were differences based on processing and variety across each stage of the experiments. Even though each product was different in composition, varietal differences were marginal in the first stages of food compositional analysis. A further evaluation of the digestible and resistant fractions of starch in stage two revealed further differences in varieties after processing. Particularly, variety *Alumaco which* distinctly showed an increase in resistant starch across most of the processing method and fried yam losing most resistant starch during processing. In the third stage of evaluation, processing substantially reflected on the rate of starch hydrolysis when pounded yam and *Amala* was compared. Also, varietal differences were more detectable using the estimated Glycemic Index (eGI) compared with the glycemic index (GI). In summary, this study provides evidence about factors such as variety and most importantly the effect of processing on starch fractions which are main determinants that affect GI and *in-vitro* starch digestibility properties of products from White Yam. Considering the diversity of varieties that exists and other products of white yam that are commonly consumed (and were not considered in this study), exploring more genetic diversity and processing methods is recommended for further studies.

## Data availability statement

The raw data supporting the conclusions of this article will be made available by the authors, without undue reservation.

## Ethics statement

The studies involving human participants were reviewed and approved by University College Hospital University of Ibadan Ethical Review Committee. The patients/participants provided their written informed consent to participate in this study.

## Author contributions

Study design and conceptualization and writing—original draft paper: TE, RS, and BM-D. Methodology: TE and BM-D. Data analysis: TE. All authors contributed to the article and approved the submitted version.

## Conflict of interest

The authors declare that the research was conducted in the absence of any commercial or financial relationships that could be construed as a potential conflict of interest.

## Publisher's note

All claims expressed in this article are solely those of the authors and do not necessarily represent those of their affiliated organizations, or those of the publisher, the editors and the reviewers. Any product that may be evaluated in this article, or claim that may be made by its manufacturer, is not guaranteed or endorsed by the publisher.
